# Pes Planus Foot among the First and Second Year Medical Students of a Medical College: A Descriptive Cross-sectional Study

**DOI:** 10.31729/jnma.6020

**Published:** 2021-04-30

**Authors:** Diwakar Kumar Shah, Sanzida Khatun

**Affiliations:** 1 Department of Anatomy, Nobel Medical College and Teaching Hospital, Biratnagar, Nepal

**Keywords:** *foot*, *foot print*, *pes cavus*, *pes planus*, *sole*

## Abstract

**Introduction:**

Foot is a complex segmented structure formed by the articulation of 26 different bones which are held together by multiple ligaments, extrinsic tendons and the intrinsic muscles of the feet. The assessment of median longitudinal arch serves as an important reference in determining the degree of pes planus or pes cavus. This study aims to find the prevalence of pes planus among the undergraduate medical students of a medical college.

**Methods:**

A descriptive cross-sectional study was carried out in the first- and second-year undergraduate medical students of a teaching hospital after taking ethical approval from Institutional Review Committee. The study was conducted from 15th November 2019 to 14th November 2020. Eighty-seven participants were involved in study using the random sampling technique. Foot prints were collected from the participants in the A4 size paper after applying ink over plantar surface of the foot. Measurements were done using the Autodesk Autocad software. Statistical Package for the Social Sciences was used. Point estimate at 95% Confidence Interval was calculated along with frequency and proportion for binary data.

**Results:**

Out of the total subjects, 14 (8.04%) (5.14-10.94 at 95% Confidence Interval) presented with flat foot. Similarly, high arched foot was seen in 29 (16.67%) of subjects whereas normal arched foot was seen in 131 (75.29%) subjects.

**Conclusions:**

From the current study we conclude that the prevalence of pes planus was slightly higher than that compared with the similar studies.

## INTRODUCTION

Foot is a complex segmented structure formed by the articulation of 26 different bones held together by multiple ligaments.^1^ The skeleton of foot is arched in such a way that both longitudinal and transverse arches are formed within the foot with the concavity directed towards the plantar surface of the foot.^2^ Pes planus (flat foot) and pes cavus (high arched foot) are the most frequently seen foot problems.^3^ These conditions can by determined by assessment of median longitudinal arch (MLA).

Not much of attention is given to the foot health status in the developing countries like Nepal. Studies have shown that the injuries pattern may vary upon the type of foot arch.^4^ Hardly a couple of studies have been conducted in Nepal regarding the foot arches.

Hence, the present study aims to determine the prevalence of Pes Planus in the undergraduate medical students of Nobel Medical College, Biratnagar.

## METHODS

A descriptive cross-sectional study was carried out in the undergraduate medical students of 1^st^ and 2^nd^ year of Nobel Medical College, Biratnagar. The study was conducted from 15^th^ November 2019 to 14^th^ November 2020.

Ethical approval was taken prior to beginning the study from the Institutional Review Committee Nobel Medical College. Written consent was obtained from all the participants involved in the study. Roy H et al from their study reported that the prevalence of flat foot was 4.9%.^5^ Using the formula,

$$\begin{array}{ll} \text{n} & ={\text{Z}}^{\text{2}}\times \text{p}\times \text{q}/{\text{e}}^{\text{2}}\\ & ={\left(1.96\right)}^{2}\times 4.9\times (1-4.9)/{\left(0.05\right)}^{\text{2}}\\ & =74 \end{array}$$

where,

n = required sample size,
Z = 1.96 at 95% Confidence Interval (CI)
p = prevalence of pes planus, 4.9%^5^
q = 1-p
e = margin of error, 5%

Thus, a total of 87 participants were involved in the study following the random sampling technique.

Foot prints were collected from the participants in the A4 size paper after applying ink over the plantar surface of the foot. To reduce the errors due to distortion while scanning the images of the foot prints, a 5cm line was drawn at the corner of the page. Measurements were done using the Autodesk AutoCAD software 2020.

Foot axis was drawn from the center of the heel to the tip of the 2nd toe and then the foot print was divided into three equal thirds excluding the toes. Using the Autodesk AutoCAD software 2020 the areas of A, B & C were calculated and then the arch index was calculated for the individual foot using the formula

AI = B/A + B + C

Where A is the area of the forefoot, B is the area of midfoot and C is the area of the heel region of the foot.

AI scores were then used to classify the foot prints into three different categories: high (≤0.21), normal (0.21 to 0.28), low (≥0.28).^6^

Subjects who were not known to any foot disease or deformities were included in the study.

Subjects who have undergone any foot surgeries or who are known cases of any foot disease were excluded from the study.

Statistical Package for the Social Sciences software was used for the data analysis. Point estimate at 95% Confidence Interval was calculated along with frequency and proportion for binary data.

**Figure 1.**
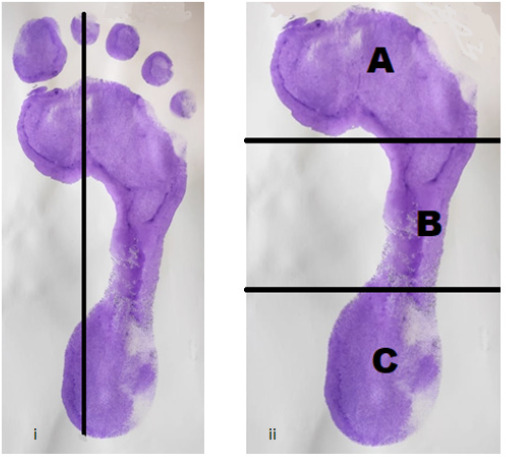
(i) Showing foot print of right foot with its central axis passing from the heel to the 2nd toe. (ii) Showing the area of Fore foot (A), Mid foot (B) and Heel region (C) of the foot.

**Figure 2.**
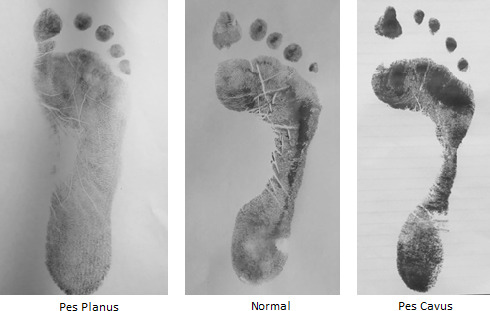
Types of foot.

## RESULTS

Out of total participants, prevalence of Pes Planus 14 (8.04%) (5.14-10.94 at 95% CI) where as 131 (75.29%) had normal arched foot and 29 (16.67%) of the subjects had high arched foot (Pes Cavus).

High arched foot was seen in 17 (19.77%) of female subjects and 11 (13.64%) of males. High arched foot was seen in the right foot in 12 (13.79%) of cases and 19.54% in the left foot. Only 8.04% of the subjects were identified with flat foot (Pes Planus) out of which 2 (2.27%) were male subjects and 12 (13.95%) were female subjects. Most of the subjects were identified with flat foot on the right side (10.34%).

Eighty-seven participants were involved in the study out of which 43 were females (49.4%) and 44 were males (50.6%).

**Table 1:** Frequency of different foot types in male and female subjects.

	Pes Cavus		Normal		Pes Planus		Total
	Left Foot n (%)	Right Foot n (%)	Left Foot n (%)	Right Foot n (%)	Left Foot n (%)	Right Foot n (%)	
Female n (%)	9 (52.94)	8 (66.667)	30 (46.15)	27 (40.9)	4 (80)	8 (88.89)	86 (49.42)
Male n (%)	8 (47.05)	4 (33.33)	35 (53.84)	39 (59.09)	1 (20)	1 (11.11)	88 (50.57)
Total	17	12	65	66	5	9	174
	29 (16.67)		131 (75.29)		14 (8.04)		

**Table 2:** Frequency of different foot types in male and female subjects.

Foot Type	Male n (%)	Female n (%)
Normal	74 (84.09)	57 (66.28)
Pes Planus	2 (2.27)	12 (13.95)
Pes Cavus	12 (13.64)	17 (19.77)
Total	88 (100)	86 (100)

**Figure 3.**
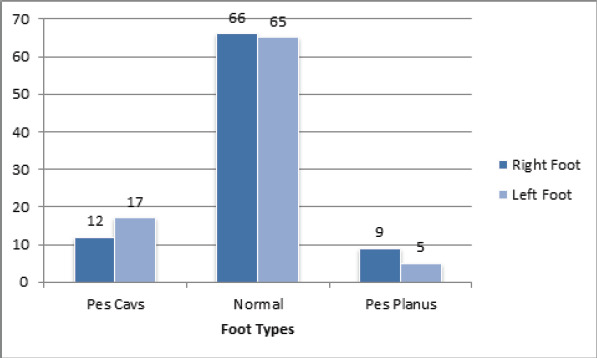
Frequency of different foot types on right and left side.

## DISCUSSION

Medial longitudinal arch remains the major component affecting the functions of the foot. For proper diagnosis and appropriate decision making regarding the reconstructive surgeries, evaluation of the foot arch remains the major component.^3^ Analysis of the foot prints for the evaluation of foot arch is the most popular technique due to its simplicity and reproducibility.^7^

The objective of the study was to determine the prevalence of pes planus in the Undergraduate Medical students of Nobel Medical College.

From the present study we found that flat foot was present in 8.04% of subjects. The results were similar and comparable to the results of many authors who from their respective studies reported 5.1%, 11.25%, 10%, 13.6%0, 16.1% prevalence of pes planus.^4, 8–11^

Vangara SM, et al. from their study reported that the overall prevalence of pes planus was 26.4% and 25.6% for right and left foot respectively.^1^ Another study done in Nigerian children by Ezema CI, et al. reported the prevalence of flat foot in 22.4% of subjects.^12^ A study carried on the Iranian students showed a very high incidence of flat foot i.e 74%.^13^ A Srilankan study reported the incidence of flat foot in 51.6% of subjects.^14^ These results were comparatively much higher than what we found from the present study.

We also found that 16.67% of the total subjects examined showed high arched foot. The findings were quite comparable with the findings of a similar study carried by Kharbuja R, et al. in the children of Bhaktapur district where they found that the high arched foot was present in 25.5% and 17.8% of subjects in the right and the left foot respectively.^4^

Pes planus may be a result of tarsal coalition, disruption of tendon of tibialis posterior, rupture of spring ligament, tarsometatarsal arthritis and hind foot degeneration or inflammatory arthritis.^15^

Erol K, et al. from their study reported that the Posterior Tibial tendon dysfunction (PTTF) remains a very important cause of Pes Planus.^16^ Apley AG in his article regarding the flat foot mentioned the anatomical and physiological causes of Pes Planus. He mentioned that the bony arch of foot is potentially unstable, bound by ligaments and is capable of resisting short term stress only. He stated that poor nervous control, inadequate muscles and infections may also cause flat foot.^17^

Vangara SV, et al. from their study reported that the incidence of Pes Cavus was 58.9% and 66.7% for the right and the left foot respectively which was very much high when compared to results from the current study.^1^

Pes cavus may arise due to some neurological disorder such as Charcot-Marie-Tooth disease, diastematomyelia, poliomyelitis etc.^15^

A large sample size can be taken in order to see the prevalence of flat foot among the Nepalese population followed by the clinical evaluation in order to establish the cause of flat foot and accordingly plan for the possible remedies/treatment for its correction.

## CONCLUSIONS

From the current study we conclude that the prevalence of pes planus was slightly higher than that compared with the similar studies.

## References

[1] Vangara SV, Gopichand PV, Bedi M, Puri N (2016). Effect of barefoot walking on foot arch structure in Tribal children. Asian Journal of Medical Sciences.

[2] Singh V (2014). Textbook of Anatomy: Abdomen and Lower Limb.

[3] Yalҫin N, Esen E, Kanatli U, Yetkin H (2010). Evaluation of the medial longitudinal arch: a comparison between the dynamic plantar pressure measurement system and radiographic analysis. Acta Orthop Traumatol Turc.

[4] Kharbuja R, Dhungel S (2017). Prevalence Of Pes Cavus And Pes Planus Among School Going Children Of Bhaktapur District, Nepal. Nepal Med Coll J.

[5] Roy H, Bhattacharya K, Deb S, Ray K (2012). Arch index: an easier approach for arch height (a regression analysis). Al Ameen Journal of Medical Sciences.

[6] Menz HB, Fotoohabadi MR, Wee E, Spink MJ (2012). Visual categorisation of the arch index: a simplified measure of foot posture in older people. Journal of foot and ankle research.

[7] Cavanagh PR, Rodgers MM (1987). The arch index: a useful measure from footprints. J Biomech.

[8] Bhoir MT Prevalence of flat foot among 18-25 years old physiotherapy students: cross sectional study.

[9] Rithanya P, Babu KY, Mohanraj KG (2018). Assessment of flat foot by plantar arch index using footprint in aged population. Drug Invention Today.

[10] Aenumulapalli A, Kulkarni MM, Gandotra AR (2017). Prevalence of flexible flat foot in adults: A cross-sectional study. J. Clin Diagn Res. JCDR.

[11] Pourghasem M, Kamali N, Farsi M, Soltanpour N (2016). Prevalence of flatfoot among school students and its relationship with BMI. Acta orthopaedica et traumatologica turcica.

[12] Ezema CI, Abaraogu UO, Okafor GO (2014). Flat foot and associated factors among primary school children: A cross-sectional study. Hong Kong Physiotherapy Journal.

[13] Askary Kachoosangy R, Aliabadi F, Ghorbani M (2013). Prevalence of flat foot: comparison between male and female primary school students. Iranian Rehabilitation Journal.

[14] Samarakoon JN, de Silva NL, Fernando D (2020). Prevalence and Associated Factors of Flat Feet among Patients with Hypertension; Findings from a Cross Sectional Study Carried Out at a Tertiary Care Hospital in Sri Lanka. Archives of Physiotherapy and Rehabilitation.

[15] Mahadevan V, Standring S. Pelvic Girdle and Lower Limb.. GRAY'S ANATOMY The Anatomical Basis of Clinical Practice.

[16] Erol K, Karahan AY, Kerimoğlu Ü, Ordahan B, Tekin L, şahin M (2015). An important cause of pes planus: the posterior tibial tendon dysfunction. Clinics and practice.

[17] Apley AG (1954). Flat foot. Postgraduate medical journal.

